# Catastrophic Bleeding From Gastroduodenal Artery After Whipple Procedure Managed With Resuscitative Endovascular Balloon Occlusion of the Aorta

**DOI:** 10.14309/crj.0000000000000283

**Published:** 2019-11-27

**Authors:** Erika Samlowski, Chris Okwuosa, Nara Tashjian, Michel Wagner

**Affiliations:** 1 Department of Surgery, Creighton University Medical Center at Bergan Mercy, Omaha, NE

## Abstract

Resuscitative endovascular balloon occlusion of the aorta (REBOA) is designed to control traumatic intra-abdominal or pelvic hemorrhage. There are few case reports of REBOA use in nontraumatic gastrointestinal (GI) hemorrhage. A 53-year-old man with pancreatic cancer status post Whipple procedure presented with GI hemorrhage from the gastroduodenal artery. Endoscopy and angioembolization were unsuccessful at stopping the hemorrhage. REBOA was used to stabilize the patient until definitive surgical control. REBOA is a potentially lifesaving measure in cases of massive abdominal or pelvic hemorrhage. REBOA can be used as an adjunct in unstable patients with GI bleeding until definitive GI, interventional radiology, or surgical control.

## INTRODUCTION

Historically, pancreaticoduodenectomy or Whipple procedure was a highly morbid operation. Advancements in surgical techniques, postoperative intensive care, and interventional radiology (IR) techniques have improved morbidity and mortality rates. Late hemorrhage, more than 24 hours after surgery, is a rare but often lethal complication after Whipple.^1,2^ Resuscitative endovascular balloon occlusion of the aorta (REBOA) is a device designed for temporary control of traumatic intra-abdominal or pelvic hemorrhage. Aortic occlusion during hemorrhage diverts blood flow to the coronary and cerebral circulation and minimizes blood loss until definitive intervention. We report a patient with massive gastrointestinal (GI) hemorrhage from the gastroduodenal artery (GDA) 2 years after Whipple, who was stabilized with REBOA.

## CASE REPORT

A 53-year-old man with a medical history of pancreatic adenocarcinoma treated with pylorus-preserving Whipple procedure 2 years before presented with hematemesis. He had symptomatic GI bleeding in the month before admission. Workup included esophagogastroduodenoscopy, colonoscopy, pill endoscopy, angiogram, and tagged red blood cell scan, but we were unable to localize the source of bleeding. He was initially hemodynamically stable, and his hemoglobin level was 7.2 g/dL. After multiple episodes of hematochezia and hematemesis, he became hypotensive and was transferred to an intensive care unit for resuscitation.

**Figure 1.**
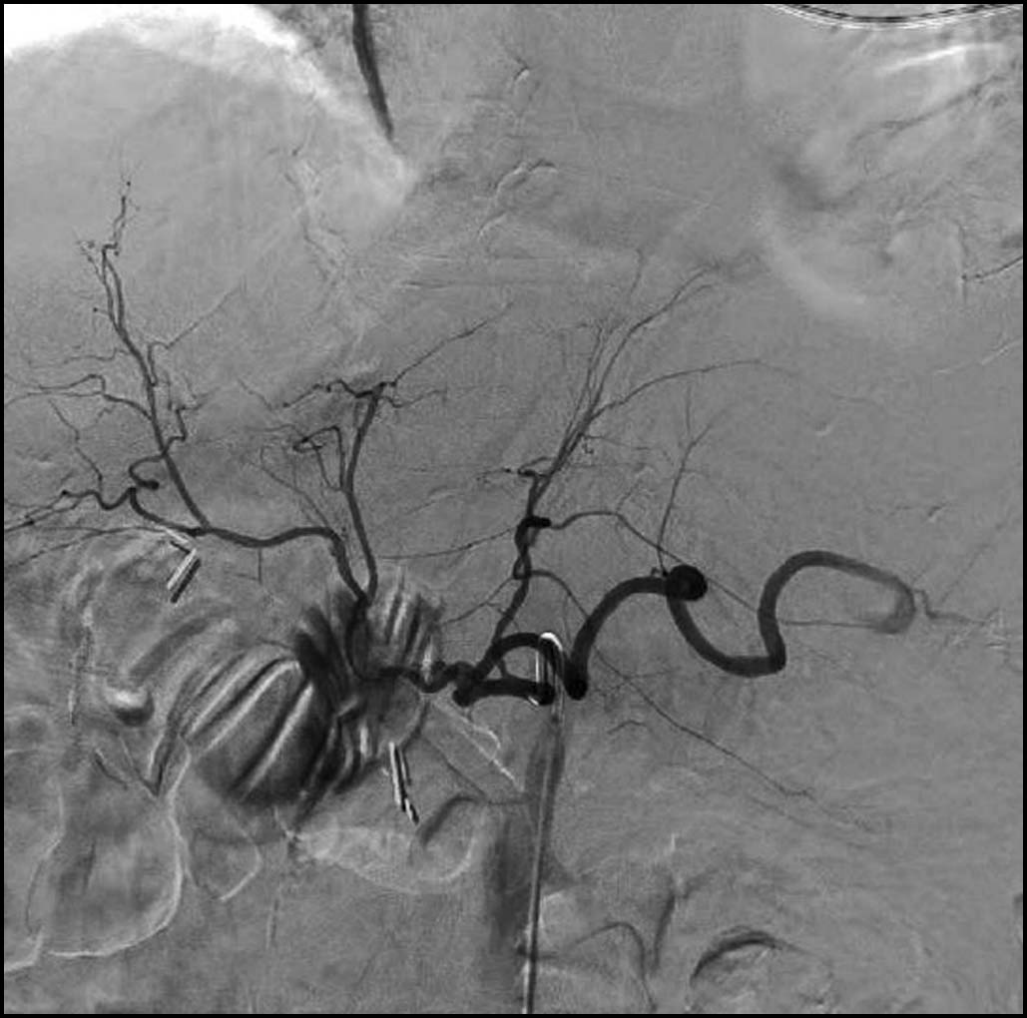
Visceral angiogram of the celiac trunk showing contrast extravasation into the lumen of the small bowel from the stump of the gastroduodenal artery.

The gastroenterology service performed emergent esophagogastroduodenoscopy with push enteroscopy, which showed a large volume of blood in the stomach, biliopancreatic limb, and efferent limb but could not localize the source of the bleeding. The patient was then taken to IR for emergent angioembolization. Angiography revealed massive active arterial bleeding directly into the small bowel from the stump of the GDA (Figure 1). Coil embolization was attempted and was unsuccessful because of brisk bleeding and a short GDA stump. At this point, the patient had lactic acidosis and hypotension requiring vasopressor support and ongoing massive transfusion necessitating emergent exploratory laparotomy.

During surgery, the patient's blood pressure stabilized, and massive transfusion was halted. Inadvertent enterotomy of the extremely dilated biliopancreatic limb during lysis of adhesions resulted in an uncontained intra-abdominal hemorrhage. The patient's blood pressure immediately dropped to 40/20 mm Hg. The patient had minimal improvement in blood pressure to 80/50 mm Hg after resuming a massive transfusion, vasopressor support, and direct aortic compression.

**Figure 2.**
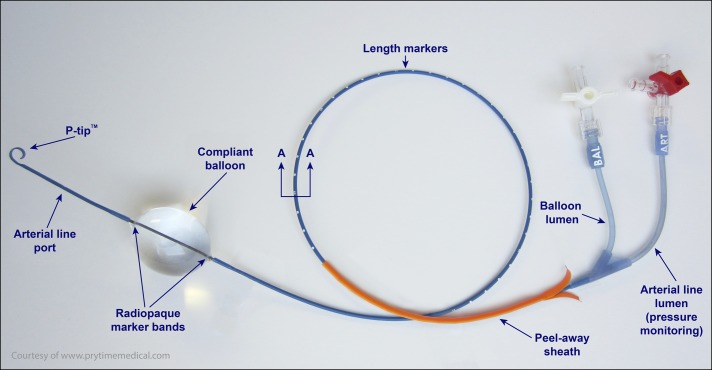
Images of REBOA device provided and reprinted with permission by Prytime Medical™, all rights reserved. ER-REBOA catheter (Prytime Medical™, Boerne, TX).

Trauma surgery placed a femoral arterial line using a standard 16-gauge catheterization kit. A guidewire was advanced through the femoral arterial line and 7 French REBOA introducer sheath placed using the Seldinger technique. Supradiaphragmatic zone 1 distance was estimated by laying the catheter from the femoral site to the xiphoid process (Figure 2). The REBOA catheter was advanced to 43 cm, and the balloon inflated with saline until blood pressure improved on radial arterial line tracing. Immediate increase in blood pressure from 60/40 to 160/90 mm Hg was seen. Vasopressor requirements decreased, and the massive transfusion protocol was able to be stopped. The REBOA balloon was deflated after 47 minutes once surgical control was obtained. Surgical blood loss was estimated more than 30 L. The patient received 130 units of blood products during surgery.

Postoperatively, the patient required minimal vasopressor support and several units of platelets. The patient had modest elevations of liver enzymes and creatinine, which resolved with continued resuscitation. The patient had no REBOA-associated femoral catheter site or limb complications. The patient underwent several subsequent abdominal washouts and definitive surgical reconstruction 9 days after his initial surgery and was successfully discharged to rehabilitation.

## DISCUSSION

Advancements in surgical technique, intensive care, and IR have significantly improved morbidity and mortality after pancreaticoduodenectomy. Morbidity rates still range from 30% to 40%, with a 5% mortality rate.^1^ Hemorrhagic complications occur in less than 10% but account for up to 44% of postoperative deaths.^1,3,4^ Early bleeding, within 24 hours of surgery, is secondary to technical failure and generally requires surgical re-exploration. Late bleeding, more than 24 hours after surgery, is secondary to pancreaticojejunostomy leak, fistula, pseudoaneurysm, or anastomotic dehiscence. Late bleeding may present with sentinel bleeding, minor GI bleeding before major hemorrhage. Concurrent sentinel bleeding with a pancreatic leak is associated with mortality rates upwards of 50%.^5^ A 2008 meta-analysis by Limongelli et al reviewed the management of delayed bleeding after Whipple.^3^ One hundred sixty-three cases were identified and the incidence of delayed bleeding was 3.9%. Presentation ranged from 5 to 206 (median 26) days after surgery. Sentinel bleeding was present in 33.1%. The underlying cause was a pancreatic leak (65.6%) and pseudoaneurysm (32.5%.) Of these patients, 47% underwent laparotomy, 45% underwent IR intervention, and 8% were managed conservatively with no statistical difference in morbidity and mortality noted.

**Figure 3.**
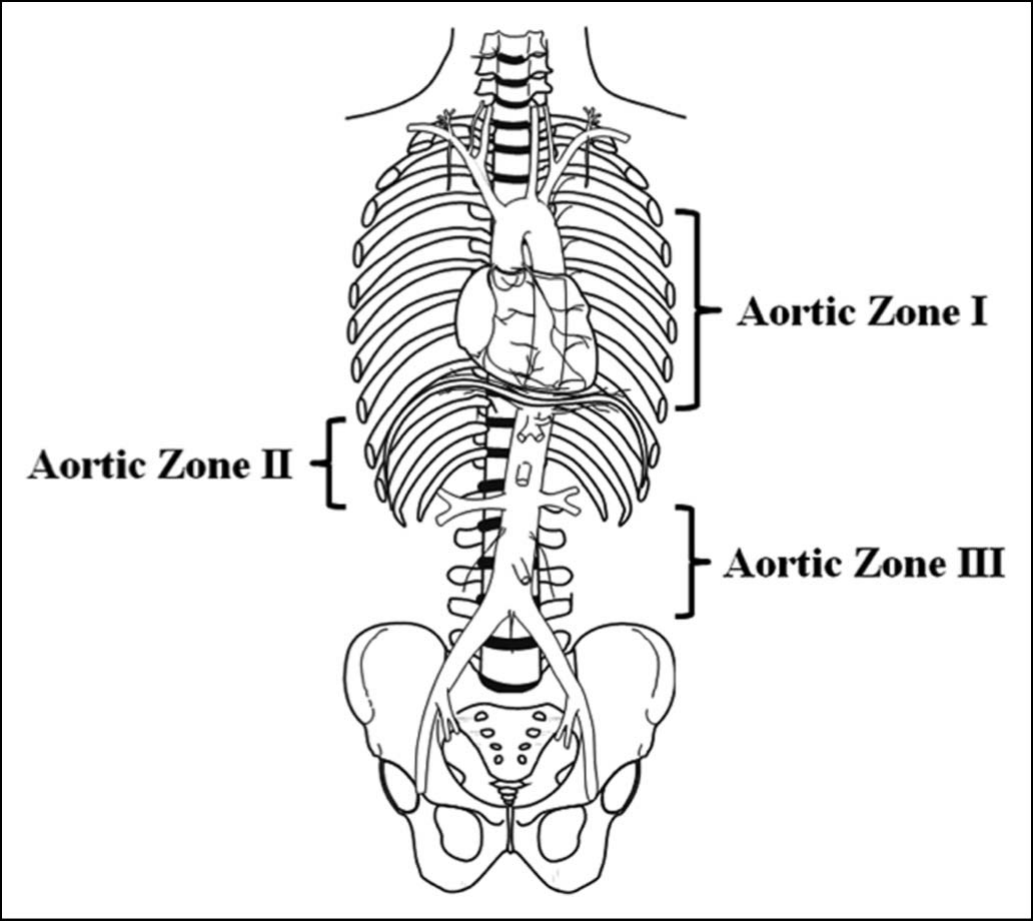
Zones for placement of REBOA. Zone 1 is between the takeoff of the left subclavian artery to the celiac trunk. Zone 2 is from the celiac trunk to the renal arteries. Zone 3 is from the lowest renal artery to the iliac bifurcation. Images reprinted with permission from Prytime Medical™.

Aortic occlusion has multiple benefits during hemorrhagic shock. It minimizes further blood loss, raises systolic blood pressure, and diverts vital blood flow to the coronary and cerebral circulation. Aortic balloon occlusion devices have existed since the Korean War. Technological advancements have allowed more widespread use in the last 10 years.^6–8^ REBOA is designed specifically for traumatic intra-abdominal or pelvic hemorrhage and has been used as an alternative to emergency department thoracotomy in unstable or arresting patients.^7^ ER-REBOA (Prytime Medical, Boerne, TX) is designed for rapid deployment in combat or trauma situations and can be introduced via femoral arterial line with a 7 French sheath without guidewire or fluoroscopy (Figure 3).

There is limited literature currently for non-trauma use of REBOA. Case reports and small series report successful utilization in ruptured abdominal aortic aneurysms, ruptured splenic artery aneurysms, postpartum hemorrhage, and aortoenteric fistulas.^9,10^ Because occlusion of the aorta in zone 1 limits blood flow to the celiac, superior mesenteric, inferior mesenteric arteries, there has been increasing interest in using REBOA for nonvariceal GI bleeding. To date, its use in this setting has largely been limited to single centers in Japan and Korea. REBOA was found to reliably improve perfusion, as reflected by systolic blood pressure, in 9 patients with gastric or duodenal ulcers when deployed in the ED, with an average aortic occlusion time of 55 minutes and 67% survival.^6^ A follow-up series of upper GI bleeding (gastric and duodenal ulcers, anastomotic bleeding, left gastric aneurysm, and esophageal cancer) in 8 patients found that REBOA could be reliably deployed and achieved improved hemodynamics until definitive control could be achieved, with a rebleeding and mortality rate both of 15%.^11^

Prolonged occlusion of the aorta, particularly in zone 1 above the diaphragm, can result in ischemia and reperfusion injuries to the kidney, bowel, and liver directly proportional to ischemia time. Metabolic derangements and lactic acidosis are common immediately after balloon deflation. Complications at the femoral artery catheter site can include pseudoaneurysm, bleeding, and limb ischemia, often seen within 24–48 hours after REBOA placement.

REBOA is a potentially lifesaving measure for abdominal or pelvic hemorrhage. We believe this patient with cataclysmic GI hemorrhage, with or without the complication of intraoperative enterotomy, would have died without REBOA intervention. This device can be used as a lifesaving bridge to definitive endoscopic, IR, or surgical management for uncontrollable nonvariceal GI bleeding of any etiology. Early use of REBOA could improve the success of GI or IR intervention and prevent patients from requiring high-risk emergent surgical intervention.

## DISCLOSURES

Author contributions: E. Samlowski wrote the manuscript. C. Okwuosa, N. Tashjian, and M. Wagner edited the manuscript. M. Wagner is the article guarantor.

Financial disclosure: None to report.

Informed consent was obtained for this case report.
